# Conversation Breeds Compliance: The Role of Intergenerational Communication in Promoting Preventive Behaviors against COVID-19 among Middle-Aged Parents in China

**DOI:** 10.3390/ijerph181910198

**Published:** 2021-09-28

**Authors:** Wanqi Gong, Qin Guo, Crystal L. Jiang

**Affiliations:** 1 School of Journalism and Communication, Guangdong University of Foreign Studies, Guangzhou 510420, China; yunkigong@gmail.com; 2 School of Humanities, Zhejiang University of Technology, Hangzhou 310023, China; menyaqin@gmail.com; 3 Department of Media and Communication, City University of Hong Kong, Hong Kong 999077, China

**Keywords:** family communication patterns, COVID-19, intergenerational communication, media exposure, middle-aged parents, preventive behaviors

## Abstract

This study aims to explore and compare the influences of two crucial information channels used by middle-aged parents—media and their adult children—on their health knowledge, emotions and preventive behaviors against COVID-19, based on media exposure and the family communication patterns (FCP) theory. Parents of college students in Guangzhou, China were invited to participate in an online survey between February 10 and 24, 2020. A total of 193 respondents, aged between 40 and 65 years, completed the study questionnaire. Media exposure was a positive predictor of negative emotions, intergenerational discussions, and preventive behaviors among Chinese middle-aged parents. Conversation orientation was a positive predictor of scientific discussions and preventive behaviors, whereas conformity orientation was a negative predictor of knowledge, but a positive predictor of intergenerational discussions and negative emotions. Intergenerational discussions mediated the relationships between media exposure and preventive behaviors, as well as between FCP and preventive behaviors. Health communication efforts require the help of adult children as intergenerational communication serves as an important amplifier in terms of influencing the health knowledge, attitudes, and behaviors of middle-aged and elderly populations.

## 1. Introduction

The middle-aged and elderly generation comprises a high-risk population for the coronavirus disease (COVID-19) pandemic [1] because they have not only suffered physical vulnerabilities, but also psychosocial stress and social isolation, during the pandemic [2]. Since, at present, there is no effective treatment for COVID-19 [3], it is important to promote preventive behaviors—maintaining social distance and wearing masks—through health communication campaigns [4,5]. Furthermore, middle-aged and elderly generations also demonstrate higher hesitancy towards receiving the COVID-19 vaccine, which is the most effective preventive measure at present [6]. Well-designed health messages and accurately targeted communication channels are two key factors for effective public health campaigns [7]. Recent studies have explored the pathologic mechanisms and psychological consequences of elderly patients [8], as well as health message strategies for COVID-19 [9], while few have explored effective health communication channels, especially for the middle-aged or older generations [10].

The Health Belief Model (HBM) points out that media and close contacts are crucial channels to provide cues for health behaviors [11,12]. The theory of planned behavior (TPB), which is also widely used in explaining health behaviors, postulates that subjective norms, attitudes, and perceived behavior control shape individuals’ behavioral intentions [13]. The subjective norms refer to one’s perceptions of certain behavior [14,15]. Significant contacts, such as family members and friends, have crucial influences on one’s subjective norms, and subsequently affect one’s health behaviors [15].

Adult children and the media are two crucial influences on the middle-aged and elderly populations’ health attitudes and behaviors [16]. Therefore, this study draws on theories of media exposure and family communication patterns (FCP) to examine the synthetic influences of two crucial information channels: the media and adult children. The findings of the study will expand our understanding of the influence of FCP on older parents’ health attitudes and behavioral intentions, and the effects of the media and family on middle-aged and elderly populations’ health attitudes and behaviors within the context of COVID-19, thus providing insights towards the promotion of the COVID-19 vaccines and the development of effective health communication strategies for middle-aged and elderly populations.

### 1.1. Media Effects on Health Prevention

The media is the main information channel of public health communication [17,18,19]. Mass media health campaigns are effective in promoting health knowledge and behaviors, such as strengthening health cognition, conveying antismoking information, reducing alcohol use, and increasing physical activities [19,20]. The rise of social media provides convenience as well as obstacles for health promotion. On one hand, since people can access and share health information with health organizations, physicians, other patients, and communities on social media [21,22], it could promote health equity among disadvantaged populations, such as low-income and rural adults [23]. On the other hand, due to the low cost of generating and disseminating information, and the lack of information gatekeepers, it could also rapidly spread health misinformation widely [24].

Media exposure to health information affects not only people’s preventive behaviors, but also influences their psychological health [25]. Scholars pointed out that repeated media exposure to COVID-19 information could cause negative emotions (e.g., anxiety, stress, etc.) and further affect physical health [26]. This effect could be more prolonged for the middle-aged and elderly generations because they are at higher risk of infection and encounter more difficulties in their daily lives as a result of social isolation. A recent study conducted in China reported that age was positively associated with negative emotions during the pandemic [27]. Based on previous literature, the following hypothesis is postulated:

**Hypothesis** **1** **(H1).** *Media exposure to COVID-19 information is positively related to middle-aged parents’: (1) knowledge, (2) preventive behaviors, and (3) negative emotions.*

Media consumption also indirectly affects people’s health attitudes, emotions, and behaviors by stimulating interpersonal communication within individuals’ social circles [7], such as parents, spouses, children, and friends. Media exposure to health campaigns sparked people’s discussions with others, and the valence of interpersonal communication affected their attitudes and self-efficacy, as well as the subjective and descriptive norms of the high-risk groups, which further influenced their preventive behaviors [28]. In general, discussions with others on media health messages generate a larger effect on their health attitudes and behaviors than exposure to media information alone. Therefore, some media health campaigns aimed at promoting health attitudes and behaviors by targeting individuals’ core network ties, such as encouraging parents to talk about sex with their children [29], emphasizing the role of husbands in safe motherhood [30], and the role of friends in adolescent smoking [31]. Thus, we propose the following hypotheses:

**Hypothesis** **2** **(H2).** *Media exposure to COVID-19 information is positively related to middle-aged parents’ discussions with their adult children.*

**Hypothesis** **3** **(H3).** *The relationship between media exposure and middle-aged parents’ emotions, COVID-19 knowledge, and preventive behaviors is mediated by their discussions with their adult children.*

### 1.2. Family Communication Patterns (FCP) and Health Behaviors

Family support—a crucial part of social support—is related to individuals’ emotions and predicted health behaviors, such as exercising, healthy eating, and diabetes self-management [32,33]. Family members also shape individuals’ subjective norms and provide cues for individuals’ health attitudes and behaviors [14,34]. Grown-up children are an important channel of interpersonal communication for older people, especially in providing and identifying health information on the Internet [35]. Intergenerational communication conducted either face-to-face or through the internet between parents and their adult children alleviates feelings of loneliness and depression, and further enhances their health and well-being [36]. Therefore, adult children play an important role in providing social support for middle-aged and elderly populations within the context of healthcare. In China, adult children and older parents are closely connected owing to its extended family model. Adults born in the 1980s and 1990s are closer to their parents because most of them are the only child in the family due to the one-child policy [16].

FCP, defined as a set of interaction schemata between parents and their children in parent–child communication [37], shape family members’ relationship perceptions and communication behaviors [38]. Family communication consists of two orientations [39]: （i) conversation orientation—the extent to which families encourage members to share ideas or feelings on different topics; and （ii) conformity orientation—the extent to which the communication environment in families emphasizes the unification of members’ values, attitudes, and beliefs [40]. It is noteworthy that FCP—either conversation or conformity oriented—are largely shaped and controlled by parents [38].

Families with high conversation orientation encourage open and equal communication and downplay the homogeneity of values and attitudes among family members, and vice versa. Previous studies found that conversation orientation was positively related to parent–child communication satisfaction, and predicted family members’ positive health attitudes and behaviors, such as sensitive health issue disclosures, mental health, and physical training [41,42]. Conversely, conformity orientation is usually linked to negative communication outcomes, including communication dissatisfaction, children’s stress, and concealment of health issues [41,42,43].

The COVID-19 pandemic broke out during the Chinese New Year holidays (24–31 January 2020) when all family members get together. The lockdown policy provided sufficient time for parent–child communication on the COVID-19 topic. Therefore, it also allowed the investigation of the influence of FCP on discussions related to infectious diseases and preventive behaviors. Previous studies concentrated more on the influence of FCP on children’s psychological well-being and health behaviors, while neglecting their influence on parents, especially older parents. We speculate that FCP, at the same time, shape the parents’ health behaviors by defining the tone and the themes of health-related discussions between parents and their adult children. More specifically, conversation orientation is expected to cultivate active parent–child communication and positive outcomes, whereas conformity orientation is expected to function in the opposite manner. Thus, we posit the following hypotheses:

**Hypothesis** **4** **(H4):** *Conversation orientation is positively related to middle-aged parents’ discussions with their adult children, their COVID-19 knowledge, and their preventive behaviors, but is negatively related to their negative emotions.*

**Hypothesis** **5** **(H5).** *Conformity orientation is negatively related to middle-aged parents’ discussions with their adult children, their COVID-19 knowledge, and their preventive behaviors, but is positively related to their negative emotions.*

**Hypothesis** **6** **(H6).** *Discussions with adult children mediate middle-aged parents’ relationship between FCP and their negative emotions, their COVID-19 knowledge, and their preventive behaviors.*

## 2. Methods

### 2.1. Recruitment

The current focus of this study—which formed part of an intergenerational health-promotion project—comprised the health-related conditions among China’s middle-aged and elderly populations, and it considered people with adult children in terms of their relationships as older parents. It was approved by the university research ethics committee. Students from two universities in Guangzhou, China were asked to invite their parents to participate in an online survey between 10 and 24 February 2020. During this period, universities in mainland China used online classes to teach and the government had appealed to the public to stay at home and maintain social distancing, which prompted the college students to live with their parents and become the primary source of interpersonal contact for their older parents. Respondents were paid 15 CNY for each questionnaire.

### 2.2. Measures

The survey items, which included the middle-aged parents’ demographics, perceived FCP, media exposure, and intergenerational discussion topics, assessed their knowledge, negative emotions, and preventive behaviors against COVID-19 during the initial outbreak. Unless otherwise specified, 5-point Likert scales, ranging from “*never*” to “*most frequently*” were used for measurements. The results were averaged to create composite scores for statistical analysis (refer to Table 1 for details).

The FCP contained two dimensions: conversation orientation and conformity orientation, and each was measured with five items adapted from Fitzpatrick and Ritchie’s Revised FCP scale [44]. Media exposure was assessed with four items (e.g., “*How often do you use media to access information about the COVID-19 trends?*”) Family discussions had two subscales: (i) scientific discussion, which was assessed with five items (e.g., *“How often do you discuss the knowledge about the disease with your family?”*); and (ii) disputed communication, which was measured with two items (e.g., *“How often do you have disagreements with your family regarding the spread of the disease?”*)

The scale that measured knowledge about COVID-19 was adapted and revised from a previous study [45]; it was evaluated with 33 true/false statements such as: *“Does wearing a mask prevent diseases?”* A higher score indicated better health knowledge on COVID-19. Four items—afraid, nervous, scared, and irritable—were adapted from the Positive and Negative Affect Schedule [46] to measure negative emotions. Preventive behaviors included two sub-dimensions: social distancing and cleanliness control. While the social distancing item inquired whether the respondents had reduced the frequency with which they left the home, avoided gatherings with family and friends, and wore a mask when going out, the cleanliness control item asked the respondents whether they had cleaned their households and been washing more often. The scale was adapted from a previous study [45].

### 2.3. Data Analysis and Model Specification

**Figure 1:**
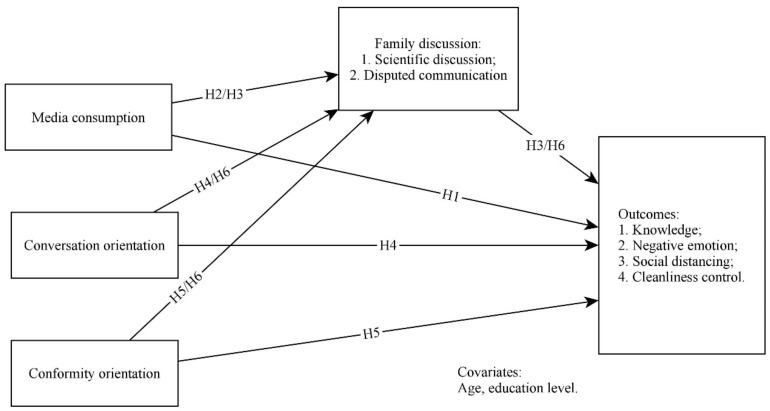
Research model.

A mediation model was constructed to test the hypotheses (Figure 1). It considered the older generation’s COVID-19-related knowledge, negative emotions, social distancing, and cleanliness control behaviors as dependent variables, their media exposure, conversation and conformity orientations, scientific discussions, and disputed communication as independent variables, and their age and education levels as covariates.

Descriptive and path analyses were conducted using IBM SPSS 26 and AMOS 24, while mediation effects were examined using Gaskin’s AMOS plugin [47]. Mediation model fit was evaluated with multiple indices: the root mean square error of approximation (RMSEA), the standardized root mean square residual (SRMR), the comparative fit-index (CFI), and the Tucker–Lewis index (TLI). The recommended values were RMSEA < 0.08, SRMR < 0.08, CFI < 0.90, and TLI < 0.90.

## 3. Results

A total of 193 respondents (85.78% response rate), aged between 40 and 65 years, of which 56% were females and 17.6% had a bachelor’s degree or above, participated in the study and completed the questionnaire (Table 2).

The overall path model demonstrated a good fit to the data: χ^2^_(17)_ = 22.177, *p* = 0.178; CFI = 0.981, TLI = 0.938; RMSEA = 0.040, SRMR = 0.0430. The non-significant relationships were removed to refine the model, including the paths from media exposure to negative emotions and knowledge, and from family communication orientations to all dependent variables. None of the removed paths resulted in significant chi-square changes. The revised model fit the data well: χ^2^_(27)_ = 36.392, *p* = 0.107; CFI = 0.966, TLI = 0.930; RMSEA = 0.043, SRMR = 0.0531.

**Figure 2:**
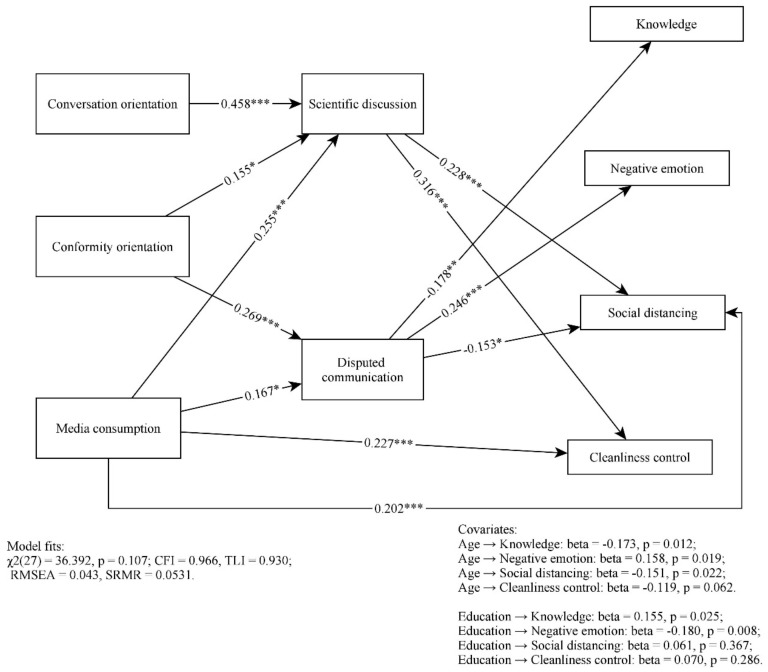
Path analysis of the hypothesized model. *** *p* < 0.001; * *p* < 0.05.

The path coefficients are presented in Figure 2. Overall, while knowledge on COVID-19, social distancing behaviors, and cleanliness control behaviors decreased with age, negative emotions increased with age. Those who were more educated were less likely to experience negative emotions and more likely to have better knowledge about COVID-19.

H1 predicted positive relationships between media exposure and the older generation’s health-related outcomes. The results suggested that media exposure was a positive predictor of negative emotions (β = 0.065, *p* = 0.010) and two preventive behaviors (for social distancing: β = 0.202, *p* = 0.008; for cleanliness control: β = 0.227, *p* = 0.003). However, the relationship between media exposure and knowledge was not significant (*p* = 0.183). Thus, H1 was partially supported.

H2 predicted a positive relationship between media exposure and intergenerational discussions. The results suggested that media exposure was positively associated with scientific discussions (β = 0.255, *p* < 0.001) and disputed communication (β = 0.167, *p* = 0.015). Thus, H2 was fully supported.

H3 hypothesized that intergenerational discussions would mediate the relationship between media exposure and the health-related outcomes of the parents. The analysis separately examined the mediation effects of scientific discussions and disputed communication (see Table 3 for indirect effects). Scientific discussions mediated the relationships between media exposure and two preventive behaviors (for social distancing: β = 0.058, *p* = 0.002; for cleanliness control: β = 0.081, *p* = 0.001). However, it did not mediate media exposure’s relationship with: knowledge (*p* = 0.995) and negative emotions (*p* = 0.178). Disputed communication mediated media exposure’s relationship with: knowledge (β = −0.030, *p* = 0.024), negative emotions (β = 0.041, *p* = 0.018), and social distancing behaviors (β = −0.026, *p* = 0.030); however, it did not mediate the relationship between media exposure and cleanliness control (*p* = 0.111).

H4 predicted the overall positive relationships between conversation orientation, intergenerational discussions, and health-related outcomes. The results suggested that conversation orientation was a positive predictor of scientific discussions (β = 0.458, *p* < 0.001) and preventive behaviors (total effect: for social distancing, β = 0.096, *p* = 0.005; for cleanliness control, β = 0.139, *p* = 0.001), but it was irrelevant to disputed communication (*p* = 0.439), knowledge (*p* = 0.792), and negative emotions (*p* = 0.125). Hence, H4 was partially supported.

H5 predicted the overall negative relationships between conformity orientation, intergenerational discussions, and health-related outcomes. The results indicated that conformity orientation was a negative predictor of knowledge (total effect: β = −0.048, *p* = 0.023), but a positive predictor of intergenerational discussions (scientific discussions: β = 0.155, *p* = 0.011; disputed communication: β = 0.269, *p* < 0.001), and negative emotions (total effect: β = 0.081, *p* = 0.001). The relationships between conformity orientation and preventive behaviors were not significant (for social distancing, *p* = 0.781; for cleanliness control, *p* = 0.450). Hence, H5 was partially supported.

H6 hypothesized that intergenerational discussions would mediate the relationship between FCP and the parents’ health-related outcomes. Two communication patterns were tested separately in terms of the mediation effects of scientific discussions and disputed communication (check Table 4 for details). For conversation orientation, scientific discussions mediated its relationships with social distancing (β = 0.104, *p* = 0.002) and cleanliness control (β = 0.145, *p* = 0.001), but did not mediate its relationship with knowledge (*p* = 0.993) and negative emotions (*p* = 0.219). Disputed communication did not mediate any paired relationship between FCP and parents’ health-related outcomes (knowledge: *p* = 0.357; negative emotions: *p* = 0.387; social distancing: *p* = 0.342; and cleanliness control: *p* = 0.321).

For conformity orientation, scientific discussions mediated its relationships with two preventive behaviors (social distancing: β = 0.035, *p* = 0.008; cleanliness control: β = 0.049, *p* = 0.009), but did not mediate its relationships with: knowledge (*p* = 0.986) and negative emotions (*p* = 0.123). Disputed communication mediated conformity orientation’s relationships with: knowledge (β = −0.048, *p* = 0.010), negative emotions (β = 0.066, *p* = 0.001), and social distancing (β = −0.041, *p* = 0.005). It also mediated the relationship between conformity orientation and cleanliness control at a marginal level (β = −0.031, *p* = 0.090). Hence, H6 was partially supported.

## 4. Discussion and Conclusions

### 4.1. Discussion

Through a survey, the study examined and compared the effects of media exposure and intergenerational discussions on middle-aged parents’ health knowledge, negative emotions, and preventive behaviors during the first phase of the COVID-19 pandemic. In China, during the Chinese Lunar New Year, many middle-aged parents went through the quarantine period with their adult children. They were exposed to a high volume of media coverage of the COVID-19 outbreak and, at the same time, engaged in extensive discussions with their adult children. Our results suggest that both media exposure and scientific discussions between parents and their adult children were positively associated with two preventive behaviors (social distancing and cleanliness control), but disputed communication between parents and their adult children was associated with less health knowledge, less compliance in social distancing, and an increase in negative emotions. Intergenerational discussions appeared to mediate the relationships between media exposure and the parents’ knowledge, negative emotions, and preventive behaviors. Specifically, scientific discussions served as a positive mediator between media exposure and two preventive behaviors, whereas disputed communication was a negative mediator between media exposure and knowledge, as well as social distancing behaviors, but a positive mediator between media exposure and negative emotions.

FCP were predictive of intergenerational discussions. Conversation orientation was associated with more scientific discussions, whereas conformity orientation was associated with more disputed communication, and, to a much lesser extent, with scientific discussions. Scientific discussions mediated the relationship between conversation orientation and two preventive behaviors, whereas disputed communication mediated the relationship between conformity orientation and some outcome variables (knowledge, negative emotions, and social distancing). These findings enrich our understanding of how family communication helps middle-aged and elderly populations acquire health knowledge and engage in preventive behaviors in public health crises. The findings can also assist public health organizations and professionals in designing tailored health promotions to influence the middle-aged and elderly populations.

This study supports the positive effects of media exposure on preventive behaviors among Chinese middle-aged parents, as well as providing evidence for the hypothesized indirect effects, whereby media exposure affects the parents’ preventive behaviors through stimulating interpersonal communication within their social networks [7]. However, in this study, media exposure to COVID-19 information had little influence on the parents’ knowledge and emotions, but paradoxically produced negative effects on knowledge acquisition, and enhanced negative emotions through disputed communication between the two generations. Future research should, therefore, investigate ways to help middle-aged and elderly populations improve their health knowledge and capacity to cope with negative emotions, provided these have enduring effects on their self-management.

As indicated by our data, intergenerational communication plays an important role in capitalizing on the parents’ media exposure to health information. In the case of COVID-19, scientific discussions, usually initiated or led by adult children, positively mediated the relationship between media exposure and preventive behaviors. In contrast, disputed communication reflected intergenerational disagreements in terms of views, and it also suppressed the capitalization of health information towards the promotion of preventive behaviors. This finding is aligned with the recent literature on reverse socialization, which is the process wherein parents learn from their adolescent or adult children about how to use smartphones or shop online. Provided that this study provides evidence of reverse socialization in the context of health communication, future research should examine reverse socialization in other health contexts, such as the prevention and treatment of chronic diseases.

This study also adds to our understanding of intergenerational communication by identifying FCP as an important antecedent for intergenerational health discussions. Conversation orientation in family communication is likely to motivate scientific discussions, which further promote two preventive behaviors. In contrast, rather than promoting scientific discussions, conformity orientation is more likely to motivate disputed communication, which further impedes knowledge acquisition, behavioral enhancements, and psychological coping. FCP essentially reflect the power dynamics in family communication. Families with high conversation orientation normally embrace openness and equality among family members; thus, the parents are more likely to appreciate their adult children’s knowledge sharing, endorse their scientific viewpoints, and follow their recommended behaviors. In the long term, conversation orientation cultivates reciprocal socialization within the family, whereby both the parents and their adult children actively engage in the sharing of knowledge and the enhancement of health. The reciprocal process also facilitates emotional bonding and enhances family solidarity and psychological health. By contrast, families with high conformity are likely to discourage value diversity and emphasize parental power. Parents may view knowledge sharing and health education as a challenge to their power. When disputes arise concerning health issues, they may disregard their children’s knowledge sharing. Their adult children may choose to disclose fewer opinions and thoughts to avoid conflicts and to maintain family harmony. As such, avoidance creates distance and detachment, thereby subjecting the parents to a more isolated status in terms of health information acquisition and self-management.

### 4.2. Conclusions

This study has examined the preventive behaviors against COVID-19 among the Chinese middle-aged and elderly populations from an intergenerational communication perspective. While the parents are exposed to the effects of media exposure and intergenerational discussions, intergenerational discussions play a crucial role by mediating the relationships between media exposure and COVID-19 knowledge, negative emotions, and preventive behaviors. Conversation orientation in family communication cultivates effective scientific discussions and breeds health compliance, while conformity orientation likely leads to disputed communication and reduces the effectiveness of health messages.

### 4.3. Practical Implications

The findings also offer two practical implications for public health professionals, middle-aged and elderly parents, and their adult children. First, since our results indicate that media exposure to health messages has a direct effect on preventive behaviors, public health professionals should make more efforts to design media health messages tailored for the targeted populations. Health message designs need to consider the middle-aged and elderly populations’ health literacy and cognitive levels and incorporate message elements that are easy for them to understand. Previous studies have suggested that several strategies (i.e., stories, visual aids, and plain language) help to enhance message processing for the elderly [48].

Second, health communication efforts require the help of adult children as intergenerational communication serves as an important amplifier in terms of influencing the parents’ health knowledge, attitudes, and behaviors. Campaigns should clearly identify adult children as key stakeholders in health enhancements that aim to reach middle-aged and elderly populations, and should also provide them with instructions on monitoring their parents’ health conditions and how they can effectively communicate with their parents to inform them regarding health decisions. Messages should also address the importance of conversation orientation in family communication, as it creates a constructive atmosphere for intergenerational bonding and health enhancement. As FCP are largely shaped and controlled by parents, parents should be instructed to take the initiative to respect different perspectives and avoid judgmental thoughts. Methods such as dyadic interviews and family consultations could help in building mutual trust and reshaping FCP.

### 4.4. Limitations

The current study has three limitations. First, its findings are limited to its study sample, which mainly comprised a relatively small sample of Chinese parents, whose average age was around 50 years. Future studies should, therefore, further test the relationships between FCP and health behaviors in older groups (e.g., parents over 60 years old) and in a wider range of cultural contexts. Second, its findings could only capture face-to-face intergenerational communication because the parents and their adult children were staying together during the Chinese Lunar New Year. The physical proximity during this special period might have biased the parents’ responses as their adult children exerted a much stronger, direct influence on their attitudes and behaviors. It is unclear whether the influences of FCP would have remained similar when both sides moved to mediated environments, especially since these situations had the potential to be different; for example, adult children could have been more distanced and would have needed to compete with other sources of influence. Third, the study did not measure vaccination attitudes and behaviors owing to the fact that COVID-19 vaccines were not available when the study was conducted. Future research should examine how intergenerational communication affects the parents’ vaccine decisions as this perspective bears important implications for the reduction in vaccine hesitancy and the promotion of vaccine uptake among middle-aged and elderly populations.

## Figures and Tables

**Table 1:** Description of scales.

	Items	Reliability	Mean ± SD
Conversation orientation	5	0.878	3.653 ± 0.689
Conformity orientation	5	0.802	3.154 ± 0.699
Information acquisition	4	0.752	4.030 ± 0.605
Scientific discussion	5	0.887	3.959 ± 0.646
Disputed communication	2	0.787	2.114 ± 0.986
Knowledge	33		28.824 ± 2.010
Negative emotions	4	0.869	2.367 ± 0.824
Precautionary measures			
Social distancing	3	0.851	4.646 ± 0.468
Cleanliness control	2	0.659	4.192 ± 0.680

**Table 2:** Proportions and means of participants.

Variable	N (%)
Gender	
Female	108 (56.0)
Male	85 (44.0%)
Age (mean ± SD)	49.18 ± 4.11
Education	
Primary school and/or below	15 (7.8%)
Middle school	49 (25.4%)
High school/vocational qualifications or equivalent	70 (36.3%)
Junior college	25 (13.0%)
Bachelor’s degree	28 (14.5%)
Master’s degree or above	6 (3.1%)
Monthly income	
Up to ¥2999	43 (22.3%)
¥3000–¥4999	48 (24.9%)
¥5000–¥9999	67 (34.7%)
¥10,000–¥19,999	20 (10.4%)
More than ¥20,000	15 (7.8%)

**Table 3:** Influence of media on elderly people’s health-related outcomes.

Paths	B	Lower	Upper	*p*	β
Indirect effect					
Media → Scientific discussion → Knowledge	0.001	−0.094	0.114	0.995	0.000
Media → Disputed communication → Knowledge	−0.099	−0.248	−0.019	0.024	−0.030 *
Total effect					
Media → Knowledge	−0.099	−0.076	0.007	0.183	−0.030
Indirect effect					
Media → Scientific discussion → Negative emotion	0.033	−0.007	0.080	0.178	0.024
Media → Disputed communication → Negative emotion	0.056	0.014	0.124	0.018	0.041 *
Total effect					
Media → Negative emotion	0.089	0.023	0.120	0.010	0.065 *
Indirect effect					
Media → Scientific discussion → Social distancing	0.045	0.020	0.084	0.002	0.058 **
Media → Disputed communication → Social distancing	−0.020	−0.049	−0.003	0.030	−0.026 *
Direct effect					
Media → Social distancing	0.156	0.084	0.350	0.008	0.202 **
Total effect					
Media → Social distancing	0.182	0.127	0.349	0.001	0.234 ***
Indirect effect					
Media → Scientific discussion → Cleanliness control	0.091	0.044	0.159	0.001	0.081 ***
Media → Disputed communication → Cleanliness control	−0.022	−0.065	0.000	0.111	−0.019
Direct effect					
Media → Cleanliness control	0.255	0.099	0.315	0.003	0.227 **
Total effect					
Media → Cleanliness control	0.324	0.179	0.399	0.001	0.289 ***

*** *p* < 0.001; ** *p* < 0.01; * *p* < 0.05.

**Table 4:** Mediation effects of family communication patterns on elderly people’s health-related outcomes.

Paths	B	Lower	Upper	*p*	β
Conversation orientation → Elderly people’s health-related outcomes					
Indirect effect					
Conversation → Scientific discussion → Knowledge	0.001	−0.158	0.168	0.993	0.000
Conversation → Disputed communication → Knowledge	−0.028	−0.133	0.023	0.357	−0.009
Total effect					
Conversation → Knowledge	−0.027	−0.064	0.050	0.792	−0.009
Indirect effect					
Conversation → Scientific discussion → Negative emotion	0.052	−0.018	0.110	0.219	0.043
Conversation → Disputed communication → Negative emotion	0.016	−0.018	0.064	0.387	0.013
Total effect					
Conversation → Negative emotion	0.067	−0.005	0.120	0.125	0.056
Indirect effect					
Conversation → Scientific discussion → Social distancing	0.071	0.037	0.121	0.002	0.104 **
Conversation → Disputed communication → Social distancing	−0.006	−0.024	0.006	0.342	−0.008
Total effect					
Conversation → Social distancing	0.065	0.044	0.165	0.005	0.096 **
Indirect effect					
Conversation → Scientific discussion → Cleanliness control	0.143	0.076	0.232	0.001	0.145 ***
Conversation → Disputed communication → Cleanliness control	−0.006	−0.031	0.005	0.321	−0.006
Total effect					
Conversation → Cleanliness control	0.137	0.071	0.219	0.001	0.139 ***
Conformity orientation → Elderly people’s health-related outcomes					
Indirect effect					
Conformity → Scientific discussion → Knowledge	0.000	−0.055	0.06	0.986	0.000
Conformity → Disputed communication → Knowledge	−0.138	−0.312	−0.040	0.010	−0.048 **
Total effect					
Conformity → Knowledge	−0.138	−0.104	−0.011	0.023	−0.048 *
Indirect effect					
Conformity → Scientific discussion → Negative emotion	0.017	−0.001	0.058	0.123	0.015
Conformity → Disputed communication → Negative emotion	0.078	0.037	0.136	0.001	0.066 ***
Total effect					
Conformity → Negative emotion	0.096	0.046	0.130	0.001	0.081 ***
Indirect effect					
Conformity → Scientific discussion → Social distancing	0.024	0.01	0.046	0.008	0.035 **
Conformity → Disputed communication → Social distancing	−0.028	−0.052	−0.011	0.005	−0.041 **
Total effect					
Conformity → Social distancing	−0.004	−0.044	0.029	0.781	−0.006
Indirect effect					
Conformity → Scientific discussion → Cleanliness control	0.048	0.019	0.095	0.009	0.049 **
Conformity → Disputed communication → Cleanliness control	−0.030	−0.069	0.000	0.090	−0.031 †
Total effect					
Conformity → Cleanliness control	0.017	−0.023	0.070	0.450	0.018

*** *p* < 0.001; ** *p* < 0.01; * *p* < 0.05; † *p* < 0.10.

## Data Availability

Not applicable.
